# Clarification of Some Bonding Concepts: Virial Theorem, Electron Pair Repulsion, and Rotational Barriers

**DOI:** 10.1002/jcc.70085

**Published:** 2025-03-28

**Authors:** W. H. Eugen Schwarz, Gernot Frenking, Sudip Pan

**Affiliations:** ^1^ Department of Chemistry University of Siegen Siegen Germany; ^2^ Theoretical and Computational Chemistry Center Tsinghua University Beijing China; ^3^ Institute of Advanced Synthesis, Nanjing Technical University Nanjing China; ^4^ Donostia International Physics Center San Sebastian Spain; ^5^ Fachbereich Chemie Philipps Universität Marburg Marburg Germany; ^6^ Institute of Atomic & Molecular Physics, Jilin University Changchun China

## Abstract

The molecular *virial theorem* relates kinetic and potential energies (*T* & *V*) to total energy and forces (*E* & *R*·∂*E*/∂*R*); it is a useful tool for analyzing the data, but does not provide clues on the origin of the stability of the “bonded” state. A strict conceptual distinction between cause and effect is recommended. Depending on the physical relationships, the induced change of one variable of the system leads to a resulting change of another variable; relaxation or response of the system can either moderate this change (in the sense of Le Chatelier's principle), enhance it, or even reverse it. Such unexpected, paradoxical behavior is common in reality and in daily life. As two examples of conceptual mix‐up in molecular chemistry, we discuss details of the origin of the steric pair‐pair repulsion and of the internal rotation barrier in ethane.

## Graphical abstract

The electrons of the overlapping, basically neutral fragments do not cause electrostatic repulsion but attraction by the pulling nuclei. The Pauli‐repulsive quantum kinetic increase of the energy density in the overlapping region always leads to reactions of the geometric structure and the electronic wave function. According to the virial theorem, this leads to a decrease in the kinetic energy value and an increase in the potential energy value, which results from the sum of large terms with opposite signs. This reaction to the physical cause does not become its physical origin, even if it is large and is repeated in the literature.

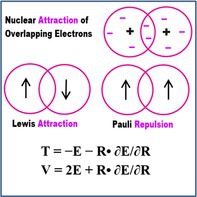

## 1 Introduction

We defend seven logically trivial and factually correct rules, initiated by conceptually problematic discussions of chemical bonding in the literature, for example, in the preceding article [1]. (1) Distinguish strictly between *cause and effect*. (2) When exploiting the molecular virial theorem, apply its *correct formula in the correct way*. (3) In addition to the classical Coulomb interactions of charge and pair densities, also consider the *quantum–mechanical motion* of the electrons. (4) *Steric repulsion of closed shells* is not because of the electrostatic attraction of overlapping charge distributions. (5) The *largest contribution of the same sign* as the total effect is not necessarily its cause. (6) Intuitive explanations require a *wise choice of reference*, which must remain within realistic limits. (7) The *relaxation of a perturbed system* can weaken (in the sense of Le Chatelier), strengthen, or even reverse the expected effect, depending on the system and on the chosen pair of causal and affected variables. The fast reader will find our main messages in the final section (Section 3).

## 2 Discussion

### 2.1 Cause and Effect

The *origin or cause* of a physical mechanism in a system leading to a specific *resulting effect or outcome* concerns two different concepts that must be distinguished. In natural science, one strives for a clear description of WHAT is in nature, and possibly one can in addition find (more than) one human explanation for *WHY* we observe that outcome.

Other philosophical standpoints are possible, and one should then stick with them [2, 3]. One option is: There are the facts and laws of nature, such as quantum theory, and they do not require further human intuitive interpretation in terms of a physical reason. Then one shall not argue that the result, nor its main contribution, is the origin (for details see below). Philosophically, this viewpoint (supported by the Bader school) avoids problematic questions about the Why; for the practice an intuitive guide for further research is lost, however [4, 5].

Another standpoint is: The transition from the initial to the final state occurs according to the mathematical laws. They are in general expressible in differential Hamiltonian form (with *H* = *T* + *V*) highlighting the causal deterministic aspect of nature, or in integral Lagrangian form (with *L* = *T*−*V*) highlighting the final teleologic (telos–aim) aspect of nature [6, 7]. Cause and result form a unity. Although the total expressions *H* and *L* govern the evolution of reality, it may nevertheless be informative to know the kinetic (*T*) and potential (*V*) contributions individually.

The laws of nature link the initial situation via the path with the aim and end of development [2, 3, 6]. We may artificially divide the total change, so that one part is large and intuitively understandable to us, and the other part is complex but small and need not be considered at the qualitative level. Such a partitioning scheme is not given by nature, but constructed by us to get the phenomenon understandable to us. There may be none, one, or several such explanatory approaches.

### 2.2 The Virial Theorem

The commutator of (*H* − *E*)*Ψ* = 0 with (*r*_*i*_·*p*_*i*_), *r*_i_ and *p*_i_ including both the electronic (*r*) and nuclear (*R*) coordinates and respective momenta [8], yields at the non‐relativistic lowest‐order Born‐Oppenheimer approximation.

(1) $$2\;T_{e}=\left\langle r\cdot \partial V/\partial r\right\rangle -R\cdot \partial E/\partial R$$

∂*E*/∂*R* is the slope of the Born‐Oppenheimer energy surface that vanishes (only) for stationary geometric structures (at local stable and metastable minima, or at transition saddle points). For pure Coulombic point‐charge interactions, $\left\langle r\cdot \partial V/\partial r\right\rangle =-V$. For extended nuclei, there are small deviations, also for most common basis sets. When applying corrections to the Coulomb potential functions such as in density functional theory (DFT), there are deviations of the order of 10^−1^, and possibly even larger additional terms if atomic effective core potentials (ECPs) are applied [9, 10, 11].

The molecular *virial theorem* (1) relates the kinetic and potential energy components (*T* & *V*) to the total *energy* and *forces* (*E* & *R*_*i*_·∂*E*/∂*R*_*i*_), “viria” meaning “forces” in Latin. The virial theorem holds for any geometric arrangement and for any type of chemical interactions. It enlightens us about *T* and *V*, if we only know the value of the essential total energy *E*. But it *does NOT provide clues as to the cause* of molecular stability. The virial theorem is not a basic principle or law of nature, but a *consequence* or corollary of basic theory, quite useful for a general check of the data before investigating the specific details of the special case.

Chemical stability and chemical reactions are determined by the thermal influences of typical environments; therefore, chemistry depends first on the electronic energies (given by the wave‐field, not just the densities). The main way to understand chemistry is the analysis of the energies—of stable bound states and of transition barrier states between them, and ideally of the entire transition path between the two stable stationary states. We do not share the opinion that the bonding analysis should be limited to the molecule itself, without reference to individually selected states such as the transition barrier states or the separated fragments or atoms in optimized or prepared states, because only with respect to them can the bond energy be defined.

Since in the ab initio all‐electron Coulombic nuclear point‐charge approach, ∂*V*(*r*)/∂*r* = −*V*(*r*)/*r* holds (but neither in DFT nor in ECP approaches), Equation (1) yields for a di‐centric case with distance *R*:

(2) $$E\left(R\right)=T_{e}\left(R\right)+V\left(R\right)$$

(2a) $$T_{e}\left(R\right)=-E\left(R\right)-R\cdot \partial E/\partial R$$

(2b) $$V\left(R\right)=+\text{2E}\left(R\right)+R\cdot \partial E/\partial R$$

These relations apply to any type of molecular or solid‐state bonding, regardless of whether its origin is covalent, ionic, correlative, metallic, dispersive, and so forth. nature. They do not hold (exactly) for the kinetic and potential energies from DFT or ECP calculations. For heavy atomic systems, one must also take the relativistic *α* ^2^·∂*E*/∂*α* ^2^ terms (*α* = fine‐structure constant) into account [12]. The virial theorem holds everywhere on the Born‐Oppenheimer energy surface, but between the stationary ground and transition states, one must not omit the *R*·∂*E*/∂*R* “force” terms.

### 2.3 Potential and Kinetic Energies

Chemical entities consist, at the atomistic level, of electrons (e) held together by nuclei (N). So, the energy consists of five terms, one attractive and four repulsive ones: The “repulsive” kinetic energies of the nuclei (*T*_*N*_) and of the electrons (*T*_e_), and the repulsive potential energies between the nuclei (*V*_NN_) and between the electrons (*V*_ee_); and the attractive energies between nuclei and electrons (*V*_Ne_). That is, all terms are positive except the last one. Slater had argued in 1933 that covalence is due to that negative term [11]. In the 1950s, within the frameworks of both the MO and the VB models, this argument was further developed that energy lowering for chemical bonding can only have one origin, namely a negative energy term, the nuclear‐electron attraction [13, 14]. The trivial idea that the total energy can also be reduced by reducing positive energy contributions remained the opinion of a minority [10, 15].

The molecular virial theorem is often used in computational chemistry in the context of chemical bonding phenomena and their potential and kinetic energy contributions. The originally thermodynamic theorem had been extended to the quantum realm by Fock in 1930 [9], and extended (including the *R*·∂*E*/∂*R* term) and applied to chemical bonding in 1933 by both “sides”: Hellmann [10] argued for a lower kinetic energy because of electron *delocalization over several atoms* as the cause of covalence, and Slater [11] argued for stronger nuclear‐electronic attraction energies because of *electron localization between the atoms*. Both can be interpreted as the physical background of Lewis sharing.

We now consider the case of a bonded molecular ground state at two equivalent conformational structures with a saddle‐point barrier transition state in between, energetically higher by Δ*E*. With the rotational barrier of ethane in mind, we take Δ*E* = +12 energy units (kJ/mol). On the basis of the virial theorem, we then know that for any type of interaction causing the barrier, the individual energy contributions will be as listed in the upper row of Table 1: *Independent of the physical cause* of the energy barrier of +12 units, the resulting electronic kinetic energy decreases by −12 units, whereas the resulting total potential energy is +24 units higher (less attractive).

**TABLE 1:** Typical changes of kinetic and potential energies and their components upon a molecular transition Δ (energies in units of kJ/mol; rounded averaged values from a dozen of quantum chemical calculations of the C—C single bond rotation of the two methyl groups in a vibrationless ethane molecule).

H_3_C 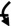 CH_3_	Δ*E* = ^a^	Δ*T*_e_+	Δ*V* =	Δ*V*_Ne_+^b^	Δ*V*_rep_ ^b^	=Δ*V*_NN_ ^b^	+Δ*V*_ee_ ^b^	*R*_*i*_·∂*E*/∂*R*_*i*_ ^a^
Relaxed structure	**+12**	**−12**	**+24**	+2A + 24 ≈ +864	−2A ≈ −840	−A − a ≈ −440	−A + a ≈ −400	**0**
Rigid rotation	**+12½**	**+25½**	**−13**	+2B − 13 ≈ −69	−2B ≈ +56	−B − b ≈ +22	−B + b ≈ +34	**−38**
Relaxation effect	−½	−37½	+37	+933	−896	−462	−434	

*Note:* Upper row: Δ from the structurally and electronically optimized molecular Ground State to the electronically optimized saddle‐point Transition State with relaxed geometric structure, higher by +12 energy units (kJ/mol). Middle row: Δ from the structurally and electronically optimized molecular Ground State to an electronically optimized state at a geometric model structure (rigid rotation with varying dihedral angles and fixed bond‐lengths and ‐angles, up to a slightly higher energy of +12½ units). The values of the energy changes in bold follow directly from the virial theorem for total energy changes Δ*E* (+12 or +12½ units). Δ*T*_*e*_ = electronic kinetic energy, Δ*V* = total potential energy, Δ*V*_Ne_ = nuclear‐electronic attraction energy, Δ*V*_rep_ = repulsive sum of Δ*V*_NN_ = nuclear‐repulsion energy, and Δ*V*_ee_ = electronic‐repulsion energy.

^a^ The experimentally derived value for Δ*E* is +12.0 kJ/mol. The reliability of the rounded averages of the computed values from our own computations and from References [1, 16, 17, 18, 19, 20, 21, 22, 23, 24] are: Of the order of ±10^0^ kJ/mol for Δ*E*, and of the order of ±10^1^ kJ/mol for the individual Δ*V* terms.

^b^ For the internal rotation of ethane, the parameters are (in kJ/mol): *A* ≈ +420 (for structurally relaxed rotation), *B* ≈ −28 (for rotation of rigid CH_3_ groups), with *a* ≈ 20 and *b* ≈ 6.

Remarkably, the potential energy increase Δ*V* of only +24 kJ/mol at the barrier saddle point is the sum of three really huge contributions Δ*V*_NN_ + Δ*V*_Ne_ + Δ*V*_ee_ = −440 + 864 − 400 (kJ/mol) of different signs and with strong cancellations. Such a situation is quite common for changes in many‐electron systems because of just slight structural and electronic relaxations. Note that changing the distance between two bound methyl groups by only about 1% (1.4 pm) already changes *V*_NN_ by about 440 kJ/mol. In such cases the questions of “why is Δ*E* positive?,” or “why is Δ*E* around 10^1^ eV?” can hardly be answered simply on the basis of the numbers describing the real process (upper row of Table 1).

Therefore, the transition from the initial optimized ground state to the final optimized saddle‐point transition state is split into two steps. The first step leads to an intermediate model state where the essential physical cause becomes apparent and a reasonable approximation for the relevant energy change is obtained. The second step then covers the major energy restructuring, but does not need to be taken into account in the essentially qualitative discussion of the origin. It has been emphasized earlier that the strategy for analyzing chemical interactions obviously has to depend on the individual case, that is, the underlying physical mechanisms in different cases only become clear when viewed from adapted perspectives and applying adapted partitioning strategies [23]. And rigid rotations of methyl groups with typical geometric structures lead to very similar barrier heights Δ*E* for every bond length in the range of typical single bonds [17].

### 2.4 Steric Repulsion

The weak, mainly attractive forces between chemical closed‐shell subsystems at larger nonoverlapping distances are due to electrostatic multipole interactions, electric induction effects, and dispersion by electron correlation. At shorter distances, the incipient charge density overlap causes additional electrostatic Coulomb *attraction* and electron‐kinematic Pauli *repulsion*, all with different radial dependences. A typical example is the increase of overlap of the bond and *tail densities* of the C—H bonds when rigid CH_3_ groups of ethane at a fixed C—C distance are rotated from staggered to eclipsed conformation (see middle row of Table 1). On average, the *H* nuclei approach a little, increasing the *V*_NN_ by Δ*V*_NN_ ≈ +22 kJ/mol; the electron–electron repulsion comparably increases by Δ*V*_ee_ ≈ +34 kJ/mol. It is rarely mentioned in the textbooks (see for example, the representative selection and references in the SI of Ref. [1]) that the increased nuclear‐electron attraction usually overcompensates the repulsion (here Δ*V*_Ne_ ≈ −69 vs. Δ*V*_rep_ ≈ +56 kJ/mol) when basically neutral electronic charge distributions around point nuclei begin to overlap. It is also rarely mentioned that the small bond orbital tails pointing toward each other in the eclipsed conformation result in similar amounts of Coulomb attraction and Pauli repulsion as the principal bond orbital densities pointing apart.

The Pauli exclusion principle forces the closed‐shell electron densities in similar spatial regions to populate higher momentum states, whereby *T*_e_ increases, here by Δ*T*_e_ ≈ +25½ kJ/mol. The Pauli exclusion also contributes to some change in the potential energy terms [17, 25]. This quantum–mechanical effective “Pauli overlap‐repulsion” overcompensates the classical electrostatic attraction energy of Δ*V* = −13 kJ/mol.

### 2.5 Not the Biggest Term Decides

Classical electron–electron repulsion was chosen as the physical origin in early intra‐atomic valence‐shell electron‐pair repulsion (VSEPR) theory, and in many general and organic chemistry textbooks for the inter‐atomic steric closed‐shell repulsion. An example is the Δ*V*_ee_ term for the *rigid* methyl rotation in ethane, which is the largest positive single term (middle row of Table 1). Bader [16] or Staemmler [1] emphasized that the small geometric and electronic response effects of the *relaxed* rotation (upper row of Table 1) change the nuclear‐electron attraction by ca. 3% more than the total Coulomb repulsion (Δ*V*_Ne_ ≈ +864 vs. Δ*V*_rep_ = Δ*V*_ee_ + Δ*V*_NN_ ≈ −840 kJ/mol). This intuitively accidental difference of Δ*V* ≈ +24 kJ/mol was then declared as the physical origin of the rotational barrier. However, it appears impossible to predict qualitatively which term is the decisive one for the sign of the sum, which is so much smaller than any of its individual contributions.

### 2.6 Appropriate Energy Partitionings

Since the basic energy terms in the expressions of the Hamiltonian or Lagrangian do not always seem to be optimal for a revealing energy partitioning, many different model partitionings have been proposed. Obviously, different molecular and reactive cases require adapted recipes that provide one (or two) decisive energy contributions of appropriate size, as well as a small remainder of large, canceling terms that need not be taken into account for an intuitive explanation. A dangerous strategy is the introduction of promoted intermediate states, whereby a small binding energy is inflated by the artificial reference.

Rotational barriers arise from the interaction of atoms and bonds between neighboring units, that is, from polycentric interactions. In such cases, polycentric MO approaches appear more appropriate than bicentric VB approaches. A single “natural resonance” Lewis‐type reference wavefunction for ethane, for example, is “promoted” by around +1.5·10^2^ kJ/mol, which is an order of magnitude larger than the rotational barrier to be explained [19, 26, 27, 28]. To approximate a realistic wavefunction, hyperconjugation was introduced and then declared as the origin of the rotational barrier. Within the framework of natural resonance theory, there is more stabilizing hyperconjugation in the staggered than in the eclipsed conformation, causing shorter and stronger C—C bonds; but there is also more Pauli repulsion in the staggered conformation [27], which sheds light on the correspondence between NRT and common chemical concepts.

### 2.7 Molecular Relaxation

The main parameter of most chemical transmutations is the distance, or the angular rearrangement between two fragments, which then usually relax geometrically during the reaction. Hence, the qualitative description of chemical transmutations usually requires two independent parameters, one describing the original initiation of the process and the other one describing the response of the system. The reaction path may be strongly bent in this coordinate system with astonishingly different variations of the internal parameters.

Many phenomena in daily life appear paradoxical or counterintuitive too. Speed limits on roads can speed up traffic flow [29, 30]; tax rate reduction can increase the state's overall tax revenue (Laffer's law) [31]; global warming because of climate change may lead to cooling in some areas; the slowing down of a satellite by the atmosphere causes it to fall down faster, and so forth. The unexpected variation of the different energy components in a chemical change, as discussed above, also falls into this general pattern, where two components of the system exhibit different dependences on an underlying internal variable that behaves according to a general law or principle, as approximated by the following simple mathematical model.

Let the basic law be that the system‐variable *E* tends to become stationary, for example, minimal (the energy) or maximal (traffic flow or tax revenue). Let *E* be the sum of two components +*T* and −*V* with different signs and different functional dependencies on some internal system parameter *p*:

(3) $$E\left(p\right)=+T\left(p\right)-V\left(p\right)$$

We use the model expressions with the model parameters *Z* and *K*:

(4a) $$-V\left(p\right)=-Z\cdot p^{1}$$

(4b) $$+T\left(p\right)=+K/2\cdot p^{2}$$

(In quantum chemistry we have the kinetic energy *T*–*p* ^2^, where p is the momentum, and the Coulomb potential energy *V*–*r* ^−1^; according to the Heisenberg uncertainty principle, Δ*r* Δ*p ≈ n* *h*; and for appropriately chosen coordinate origins, Δ*r* = ⟨*r*⟩, Δ*p* = ⟨*p*⟩, ⟨*r*⟩^−1^ ≈ ⟨*r* ^−1^⟩ ≈ ⟨*p* ^1^⟩/*n*) [15, 16, 32, 33]. The minimum of *E* is obtained for:

(5) $$\partial E\left(p\right)/\partial p=+K\cdot p-Z=0,\text{that is},p_{\mathrm{opt}}=Z/K,$$

giving the relaxed optimized values.

(6) $$+T_{\mathrm{opt}}=+½\cdot Z^{2}/K,-V_{\mathrm{opt}}=-1\cdot Z^{2}/K,E_{\mathrm{opt}}=-½\cdot Z^{2}/K,$$

what corresponds to the virial theorem.

We now assume a modified system with modified model parameters *Z* and *K*:

(7a) $$Z^{\mathrm{mod}}=Z\cdot \left(1+\zeta \right)$$

(7b) $$K^{\mathrm{mod}}=K\cdot \left(1-\kappa \right)$$

giving the relaxed optimized values of the modified system, *V*_opt_^mod^ and *T*_opt_^mod^:

(8a) $${V_{\mathrm{opt}}}^{\mathrm{mod}}=V_{\mathrm{opt}}\;{\left(1+\zeta \right)}^{2}/\left(1-\kappa \right),\Delta V_{\mathrm{opt}}\approx \left(2\zeta +\kappa \right)\;V_{\mathrm{opt}}$$

(8b) $${T_{\mathrm{opt}}}^{\mathrm{mod}}=T_{\mathrm{opt}}\;{\left(1+\zeta \right)}^{2}/\left(1-\kappa \right),\Delta T_{\mathrm{opt}}\approx \left(2\zeta +\kappa \right)\;T_{\mathrm{opt}}$$

We emphasize four points that apply to any real system in which two directly observable properties depend on two internal variables in the way as modeled here:

- The two opposing contributions *T* and *V* in Equation (3) change both upon relaxation (Equations 8a and 8b) even when only one individual parameter *ζ* or *κ* is changed.
- Although the modification (Equation 7a) increases the *V function* (Equation 4a) by a factor of (1 + *ζ*), the optimized *relaxed Vvalue* is increased to the lowest order of approximation by a factor of (1 + 2*ζ*), see Equation (8a), so to say an “over‐LeChatelier” behavior.
- Although the modification (Equation 7b) reduces the *T function* (Equation 4b) by a factor (1−*κ*), the optimized *relaxed T value* is increased, to lowest order of approximation, by a factor of (1 + *κ*), see Equation (8b), so to say an “anti‐LeChatelier” behavior.
- The final, relaxed *numerical values* of the components of the system do *not* vary (not even in the lowest order, as one might naively expect) as the parameters that describe the *functional dependence* of the components.

## 3 RÉSUMÉ

### 3.1 Summary

The present discussion was triggered by claims in the recent literature on ethane, which are here refuted.

Claim 1 The physical reason for the observed “steric” repulsion in chemistry between neutral fragments with closed shells, such as the two rotating methyl groups in H_3_C—CH_3_ (or the two noble gas atoms in Ar_2_, or the electron pairs in the valence shell around an atom) is the classical electrostatic Coulomb repulsion of the outer electron density shells. This is factually wrong.

At least since Heitler and London 1927, chemists should know that the overlap of the electronic *densities* of two neutral spherical atoms (H or He) causes a *slight reduction* of the *classical* electrostatic *potential energy* (since inside the electronic charge distributions, the nuclear attraction is incompletely shielded). However, due to *quantum–mechanical* constructive and destructive interactions of the *wavefunctions* (contra‐gradient and noncontra‐gradient overlaps) two very different *kinetic energies* result, which lead to bonding and antibonding one‐electron states. This together with the *Pauli exclusion principle* leads to covalent bonding or steric Pauli‐repulsion, depending on the number of electrons and the type of spin coupling [34, 35, 36]. Steric repulsion is dominantly determined by quantum dynamics, not by electro‐statics.

Claim 2 The physical reason for the observed rotational barrier of ethane is the reduced negative potential energy that develops for the 1% longer C—C bond in the eclipsed compared to the staggered conformation. This is a logical error.

For both shorter and longer C—C distances, the optimized rotational conformation is the *staggered one*, whereas for both staggered and eclipsed conformations, the optimized C—C distances are (slightly) different. Hence, the rotation angle is the independent reference variable between the stationary ground and barrier states. In contrast, the slightly different C—C *bond lengths* in the two conformations are a *small side‐effect* because of structural‐electronic relaxation, giving rise to stationary minimum and saddle‐point states. The total relaxation energies need not be considered in qualitative explanations, because they are an order of magnitude smaller than the barrier heights, whereas the individual canceling terms are even more than an order of magnitude bigger.

Claim 3 The physical reason for the observed “steric” repulsion in eclipsed vs. staggered ethane at +12 kJ/mol higher energy is that the nuclear‐electronic attraction energy at optimized geometries is more than 850 kJ/mol lower, being incompletely compensated by the electron–electron and nuclear‐nuclear repulsions of together only a bit more than 800 kJ/mol. This is conceptually incorrect.

First there is no obvious conclusive reason why the change of the sum of *repulsive Coulomb energy terms must be a few % smaller than the attractive component*; and second the change in *kinetic energy of similar order* of magnitude also contributes, and must not be excluded from the discussion.

Claim 4 The physical reason for the observed “steric” repulsion in the eclipsed conformation of ethane is the more pronounced electronic configuration mixing (“hyperconjugation or orbital‐mixing”), if a basis of simple valence‐bond‐NBO configurations is applied. This is another conceptual error.

The observed transition effect is here not described as a difference between the two real stationary states, but with reference to seriously simplified models, where the *model corrections for the energy terms are again an order of magnitude larger* than the effect itself.

### 3.2 Conclusions

Stability and reactivity of chemical substances depend on thermodynamic energy relations. The description of the detailed energy components depending on the geometric structures is basic. The virial theorem is helpful in this context, provided it is applied correctly, namely, including the force term (except at stationary energy points) and, where necessary, the correction terms for density functionals, ECPs, or relativity. Of course, the detailed description of what is in nature is not the origin of why it must appear that way to us. The virial theorem helps to describe it, but it does not explain it.

To answer the “why” one needs an intermediate model state between the actual initial and final states that rather simply yields an acceptable agreement of the overall properties of geometry and energy and thereby generates an explanation and supplies the reason. The transition from the approximate model to the optimized state (the only reality that exists) [5] can cause large changes of opposite sign in the various individual energy components. Therefore, their discussion is not helpful in terms of a qualitative explanation of the physical origin.

This situation may leave room for alternative, equally valid explanations. A popular conceptual mistake is the formulation: Since my explanation is acceptable, your alternative explanation is wrong. There are many molecular properties that can be expressed as derivatives of two variables. Within the framework of double perturbation theory, there are then *always* at least two different complementary explanations [37].

Direct comparison of the real stationary states of chemical transmutations seems theoretically sound; however, it does not necessarily supply intuitive insight. Due to internal geometric and electronic relaxations, the different energy contributions can vary greatly, so there is no direct explanation for why the observable sum of the terms has a positive or negative sign. The origin and cause of some outcomes may be due to an increase in *variable a*, but relaxation may increase, decrease, or even invert the *value of a*.

## Data Availability

Data sharing not applicable to this article as no datasets were generated or analysed during the current study.

## References

[1] V. Staemmler and R. Franke , “On the Origin of the Rotational Barrier in Ethane,” Journal of Computational Chemistry (2024), Preceding Paper in This Issue. 10.1002/jcc.70014 40153476

[2] M. Bächtold , “Interpreting Quantum Mechanics According to a Pragmatist Approach,” Foundations of Physics 38 (2008): 843–868.

[3] C. Friebe , M. Kuhlmann , H. Lyre , P. M. Näger , O. Passon , and M. Stöckler , The Philosophy of Quantum Physics (Springer, 2017).

[4] R. F. W. Bader , Atoms in Molecules: A Quantum Theory (Oxford University Press, 1994).

[5] R. F. W. Bader , “Pauli Repulsions Exist Only in the Eye of the Beholder,” Chemistry—A European Journal 12 (2006): 2896–2901. 16528768 10.1002/chem.200501589

[6] V. Hirvonen , “Lagrangian vs. Hamiltonian Mechanics,” (2023), https://profoundphysics.com/.

[7] M. G. Calkin , Lagrangian and Hamiltonian Mechanics (World Scientific, 1996).

[8] E. Weislinger and G. Olivier , “The Classical and Quantum Mechanical Virial Theorem,” International Journal of Quantum Chemistry 8 (1974): 389–401.

[9] V. Fock , “Bemerkung zum Virialsatz,” Zeitschrift für Physik 63 (1930): 855–858.

[10] H. Hellmann , “Zur Rolle der Kinetischen Elektronenenergie für Die Zwischenatomaren Kräfte,” Zeitschrift für Physik 85 (1933): 180–190.

[11] J. C. Slater , “The Virial and Molecular Structure,” Journal of Chemical Physics 1 (1933): 687–691.

[12] A. Rutkowski , W. H. E. Schwarz , and R. Kosłowski , “Relativistic Virial Theorem for Diatomic Molecules,” Theoretica Chimica Acta 87 (1993): 75–87.

[13] J. Lennard‐Jones and J. A. Pople , “The Molecular Orbital Theory of Chemical Valency,” Proceedings of the Royal Society of London. Series A: Mathematical and Physical Sciences 210 (1951): 190–206.

[14] R. McWeeny , “The Valence Bond Theory of Molecular Structure,” Proceedings of the Royal Society of London. Series A: Mathematical and Physical Sciences 223 (1954): 63–79.

[15] K. Ruedenberg , “The Physical Nature of the Chemical Bond,” Reviews of Modern Physics 34 (1962): 326–376.

[16] R. F. W. Bader , J. R. Cheeseman , K. E. Laidig , K. B. Wiberg , and C. Breneman , “Origin of Rotation and Inversion Barriers,” Journal of the American Chemical Society 112 (1990): 6530–6536.

[17] F. M. Bickelhaupt and E. J. Baerends , “The Case for Steric Repulsion Causing the Staggered Conformation of Ethane,” Angewandte Chemie, International Edition 115 (2003): 4315–4320, *Angewandte Chemie*, *International Edition* 42: 4183–4188. 10.1002/anie.200350947 14502731

[18] Y. R. Mo , W. Wu , L. C. Song , M. H. Lin , Q. Zhang , and J. L. Gao , “The Magnitude of Hyperconjugation in Ethane,” Angewandte Chemie, International Edition 43 (2004): 1986–1990. 15065281 10.1002/anie.200352931

[19] Y. R. Mo and J. L. Gao , “Theoretical Analysis of the Rotational Barrier of Ethane,” Accounts of Chemical Research 40 (2007): 113–119. 17309192 10.1021/ar068073w

[20] Y. R. Mo , “The Block‐Localized Wavefunction (BLW) Perspective of Chemical Bonding,” in The Chemical Bond, ed. G. Frenking and S. Shaik (Wiley‐VCH, 2014).

[21] X. Chang , P. F. Su , and W. Wu , “Internal Rotation Barrier of the XH3‐YH3 (X, Y = C or Si) Molecules,” Chemical Physics Letters 610 (2014): 246–250.

[22] M. Baranac‐Stojanović , “Theoretical Analysis of the Rotational Barrier in Ethane: Cause and Consequences,” Structural Chemistry 26 (2015): 989–996.

[23] T. Bitter , K. Ruedenberg , and W. H. E. Schwarz , “Toward a Physical Understanding of Electron‐Sharing Two‐Center Bonds. I. General Aspects,” Journal of Computational Chemistry 28 (2006): 411–422. 10.1002/jcc.20531 17143871

[24] J. Poater , D. M. Andrada , M. Solà , and C. Foroutan‐Nejad , “Path‐Dependency of Energy Decomposition Analysis & the Elusive Nature of Bonding,” Physical Chemistry Chemical Physics 24 (2022): 2344–2348. 35018916 10.1039/d1cp04135e PMC8790740

[25] F. M. Bickelhaupt and E. J. Baerends , “Kohn‐Sham Density Functional Theory: Predicting and Understanding Chemistry,” Reviews in Computational Chemistry 15 (2000): 1–86.

[26] F. Weinhold , “A New Twist on Molecular Shape,” Nature 411 (2001): 539–541. 10.1038/35079225 11385553

[27] V. Pophristic and L. Goodman , “Hyperconjugation Not Steric Repulsion Leads to the Staggered Structure of Ethane,” Nature 411 (2001): 565–568. 11385566 10.1038/35079036

[28] A. E. Reed and F. Weinhold , “Natural Bond Orbital Analysis of Internal Rotation Barriers and Related Phenomena,” Israel Journal of Chemistry 31 (1991): 277–285.

[29] B. Bidoura Khondaker and L. Kattan , “Variable Speed Limit: A Microscopic Analysis in a Connected Vehicle Environment,” Transportation Research Part C: Emerging Technologies 58A (2015): 146–159.

[30] K. Kušić , E. Ivanjko , M. Gregurić , and M. Mileti , “An Overview of Reinforcement Learning Methods for Variable Speed Limit Control,” Applied Sciences 10 (2020): 4917.

[31] M. Trabandt and H. Uhlig , “The Laffer Curve Revisited,” Journal of Monetary Economics 58 (2011): 305–327.

[32] T. Bitter , S. G. Wang , K. Ruedenberg , and W. H. E. Schwarz , “Toward a Physical Understanding of Electron‐Sharing Two‐Center Bonds. II. Pseudo‐Potential Based Analysis of Diatomic Molecules,” Theoretical Chemistry Accounts 127 (2010): 237–257.

[33] K. Ruedenberg , “Atoms and Interatomic Bonding Synergism Inherent in Molecular Electronic Wave Functions,” Journal of Chemical Physics 157 (2022): 024111, Atoms and Bonds in Molecules as Synergisms of Interactions Between Electrons and Nuclei, *Journal of Chemical Physics* 157: 210901. 35840378 10.1063/5.0094609

[34] C. W. Wilson and W. A. Goddard , “The Role of Kinetic Energy in Chemical Binding,” Theoretica Chimica Acta 26 (1972): 195–210.

[35] R. l. Snow and J. l. Bills , “Textbook Error 117—Pauli Principle and Electronic Repulsion in Helium,” Journal of Chemical Education 51 (1974): 585–586.

[36] W. J. Mullin and G. Blaylock , “Quantum Statistics: Is There an Effective Fermion Repulsion or Boson Attraction?,” American Journal of Physics 71 (2003): 1223–1231.

[37] J. Autschbach and T. Ziegler , “Double Perturbation Theory: A Powerful Tool in Computational Coordination Chemistry,” Coordination Chemistry Reviews 238/239 (2003): 83–126.

